# Twitter as a Knowledge Translation Tool to Increase Awareness of the OpenHEARTSMAP Psychosocial Assessment and Management Tool in the Field of Pediatric Emergency Mental Health

**DOI:** 10.7759/cureus.27597

**Published:** 2022-08-02

**Authors:** Alaina Chun, Rikesh Panchmatia, Quynh Doan, Garth Meckler, Badrinath Narayan

**Affiliations:** 1 Pediatric Emergency Medicine, British Columbia (BC) Children's Hospital, Vancouver, CAN; 2 Family Medicine, University of Calgary, Vancouver, CAN

**Keywords:** covid-19, evidence based management, social media, twitter, knowledge translation, mental health, emergency, pediatric

## Abstract

Rationale

The increasing prevalence of pediatric mental health presentations in pediatric emergency departments (PED) requires improved integration of evidence-based management strategies. Social media, specifically Twitter, has shown potential to aid in closing the knowledge translation (KT) gap between these evidence-based management strategies and pediatric emergency medicine (PEM) providers.

Aims and objectives

The primary outcome of this study is to evaluate the effectiveness of Twitter as a KT dissemination tool in PEM. The exploratory outcomes were to assess how to effectively implement Twitter in KT, explore ways in which Twitter can maximize the global reach of OpenHEARTSMAP and whether Twitter can lead to increased adoption of OpenHEARTSMAP.

Methods

A one-week prospective promotion on Twitter was conducted to disseminate the OpenHEARTSMAP tool using 15 topic-related hashtags (arm 1, 15 Tweets) versus one post wherein 15 different Twitter users were mentioned in 15 different comments (arm 2, 1 Tweet). A one-week control period immediately prior to posting was employed for comparisons.

Results

During the Twitter week, visits per day to OpenHEARTSMAP increased by 175%; mean time spent on the website increased by 212%; and mean page actions per visit increased by 130%. The greatest increase in visits occurred on the first day of Tweeting. Arm 2 received the greatest engagements. Within arm 1, the category of pediatrics received the most engagements (hashtag #Peds was most popular). Arm 1 received 455 impressions compared to 2071 in arm 2. No new users registered an account on the OpenHEARTSMAP website, which is required to physically use the tool.

Conclusion

Twitter can be an effective KT tool to increase awareness of research, the first step of KT, in the domain of PEM mental health care. Strategies for success include building a robust Twitter following; posting during peak healthcare-related Twitter traffic times; employing hashtags coinciding with current events; and targeting posts by tagging users who need not necessarily be generally well-known opinion leaders.

## Introduction

Knowledge translation (KT) and the KT gap

While scientific evidence has expanded over the past few decades, the gap between knowledge and action in all areas of medicine has widened. Reports have described a lag of approximately 17 years between research publication and implementation, with only 14% of new discoveries reaching bedside practice [1,2]. This gap has been coined as the KT ‘chasm.’ For this reason, up to 40% of children fail to receive appropriate evidence-based treatments in pediatric emergency departments (PEDs) [3].

The immense volume of published research coupled with limited resource access and time constraints have been cited as barriers to evidence uptake. This contributes to poor awareness, or knowledge, of published literature - the critical first step in KT [4]. Attitudes and behavioural changes once the knowledge is acquired form the subsequent steps in KT [4].

The role of social media in KT

Social media is a collection of online websites and applications where “users contribute, retrieve, and explore content generated by fellow users…for users, thus allowing knowledge and support to flow more effectively” [5]. In contrast to the static flow of information through medical journals, Tunnecliff et al. among others have validated the perception of social media in health care as a powerful, globally accessible, convenient, and cost-effective communication tool that ‘pushes’ knowledge to end-users, providing a possible avenue to fill in components of the KT gap [6,7].

Twitter in KT

Twitter is considered the most popular social media platform for healthcare communication [8]. Choo et al. commented on Twitter’s advantages as a method for KT: broad-reaching knowledge exchange; increased access to information to improve quality of care; and a platform to foster frequent collaboration and a sense of community through two-way communication [8].

Emergency medicine has been a pioneer for free open-access medical education (FOAMed) [9]. Twitter has been central to FOAMed’s development. In a survey of Canadian emergency medicine practitioners, 99.5% of residents described using FOAMed resources for general emergency medicine education. Furthermore, pediatric-specific FOAMed resources have been developed - an important step towards the translation of PEM research [10,11].

Pediatric emergency medicine (PEM) mental health and HEARTSMAP

Currently, as many as one in five children and adolescents suffers from a neuropsychiatric condition [12]. Pediatric mental health emergencies represent a significantly increasing component of PEM, as EDs become a safety net for psychiatric presentations in children [13-15]. However, ED providers have been shown to exhibit limited knowledge of effective ED psychiatric care for youth, over-reliance on consultants in the context of already reduced inpatient services, uncertainty around available mental health referral resources, and a lack of routinely used mental and behavioural health screening tools, resulting in variable and unreliable assessments [13, 15-17].

Accordingly, researchers have recommended the need for a standardized mental health-screening questionnaire with corresponding follow-up or discharge instructions [13,17,18]. To address this recommendation, our team at BC Children’s Hospital created the HEARTSMAP assessment and disposition tool (Appendix). This tool contains 10 sections (corresponding to the acronym ‘HEARTSMAP’) to help facilitate a full but efficient emergency psychosocial assessment: Home, Education and activities, Alcohol and drugs, Relationships and bullying, Thoughts and anxiety, Safety, sexual health, Mood and behaviour, Abuse, and Professionals and resources. Each section is scored from 0 to 3 with zero meaning no concerns and 1 to 3 meaning mild, moderate or severe concerns, respectively. Based on the total score, recommendations are generated to enable ED clinicians to reliably manage emergency pediatric psychosocial presentations. Potential management suggestions range from acute psychiatric consultation to outpatient referral to a crisis response team, social services, or adolescent health [19]. HEARTSMAP has shown to be a valid and reliable mental health-screening tool, with strong inter-rater agreement [20]. Although local translational efforts of this tool have been partially effective, formal evaluation revealed a need for broader dissemination.

Research gap informing this study

KT in PEM is an emerging field and there is a paucity of literature as to specific effective methods to disseminate PEM knowledge, which is the first step of KT [21]. To our understanding, no previous study has evaluated the role of social media in pediatric emergency care of mental health presentations. We sought to determine whether Twitter could effectively disseminate and increase awareness of the HEARTSMAP tool, which is specifically relevant to PEM.

Study aims

A one-week prospective social media promotion was conducted with a primary aim of determining the effectiveness of Twitter as a KT dissemination method in PEM in increasing awareness of the HEARTSMAP tool.

Exploratory aims were to determine which Twitter implementation strategy leads to maximal end-user interaction; to determine which Twitter implementation strategy leads to maximal global reach of HEARTSMAP; and to determine whether Twitter leads to increased use of HEARTSMAP.

Awareness, the primary aim of this study, forms the first component of the KT pathway, while adoption forms the last step of KT [4]. Implementation and reach were metrics used to compare the efficacy of the two study arms as described below.

## Materials and methods

OpenHEARTSMAP

To broaden the global reach and utility of HEARTSMAP, a publicly accessible version called OpenHEARTSMAP was created. The tool is available at https://openheartsmap.ca. Clinicians can register online at no cost to become an OpenHEARTSMAP user after submitting basic professional demographic information. Complete access to the tool requires successful completion of three training cases embedded within the registration process.

Study intervention

Using our established @HEARTSMAP_ Twitter account with 52 followers, 15 hashtag-specific Tweets (arm 1) and one generic Tweet without hashtags in which 15 different comments were made tagging 15 different users (arm 2) were posted as two KT dissemination strategies. All Tweets were written in less than 280 characters and contained: a traceable link to OpenHEARTSMAP, a visual component to increase dissemination and a call to action for viewers to retweet our post [22, 23]. Comments (Twitter posts tagging specific users) contained a message requesting the tagged user to retweet the generic Tweet. One Tweet was posted every 30 minutes in random order to prevent a flood of Tweets to users’ news feeds. No other randomization was used in this study. All Tweets were posted on May 6th, 2020. Data for the Twitter week ended on May 13th, 2020. The control week spanned from April 28th to May 5th, 2020.

Study arms

In arm 1, the 15 hashtag-specific Tweets referenced five different topic categories related to OpenHEARTSMAP as well as current events (COVID-19, related mental health events) to determine whether hashtag use specific to a particular period of time is influential: FOAMed, pediatrics, emergency medicine, mental health and COVID-19, with three Tweets per category (Popular FOAMed groups were identified using the latest Social Media index rankings [24]). COVID-19 was included as a category given the beginning of the pandemic was March 2020. During this time, social media was an important platform to convey information relating to school closures, isolation practices and possible effects on child and adolescent mental health [25]. The other hashtags were identified using previously published blog surveys of popular corresponding hashtags. The selected hashtags were (in order of decreasing popularity within each category): #FOAMed, #FOAMped and #MedEd (FOAMed); #Pediatrics, #Peds and #Kids (pediatrics); #emergencymedicine, #emergencydepartment and #ems (emergency medicine); #mentalhealth, #mentalhealthawareness and #mentalhealthawarenessmonth (mental health); and #COVID19, #coronavirus, #pandemic (COVID-19).

In arm 2, one generic Tweet containing our message without hashtags was tagged to 15 different users who fall into five different topic categories of accounts related to OpenHEARTSMAP: FOAMed accounts, pediatric opinion leaders, emergency medicine opinion leaders, mental health opinion leaders or organizations, and COVID-19-related opinion leaders, with three tags per category. FOAMed accounts were identified as described above. Opinion leader accounts or organizations were identified upon consultation with experienced physicians at our hospital. These accounts were identified based upon their popularity (number of followers) and/or the opinion leaders’ personal knowledge of influential individuals and organizations in the field of emergency medicine. The selected accounts were: @LITFLblog; @emcrit and @ALiEMteam (FOAMed); @dzungxvo-Dr. Dzung X Vo., @offcall99-Dr. Badrinath Narayan, @QdQwerty-Dr. Quynh Doan (pediatric opinion leaders); @Tingdan-Dr. Danial Ting; @Brent_Thoma-Dr. Brent Thoma, @TChanMD-Dr. Theresa Chan (emergency opinion leader); @Tylerblack23-Dr. Tyler Black, @Skye_Barbic-Skye Barbic and @CMHA_NTL (mental health opinion leaders); and @picardonhealth-Andre Picard, @adriandix-Adrian Dix and @drsanjaygupta-Dr. Sanjay Gupta (COVID-19-related opinion leaders).

Sample tweet

OpenHEARTSMAP makes it easier for any provider, anywhere, to assess psychosocial presentations amongst children and youth in the emergency department, especially during this pandemic. Please retweet to spread the word! (#____) Openheartsmap.ca

Sample tagged post

(@____) We considered the OpenHEARTSMAP tool, created by physicians at British Columbia's Children's Hospital in Vancouver, might be of interest to you during these times. Please do retweet this post to help spread the word, thank you!

Outcome measures

During the one-week promotion, daily metrics were collected from Twitter’s analytic tool and from the OpenHEARTSMAP web analytics page.

Primary Outcome - Awareness

The effectiveness of Twitter as a knowledge dissemination tool to increase awareness was measured by the total number of ‘page visits’, ‘average visit duration’ and ‘page actions’ on the OpenHEARTSMAP webpage. The method of access to the OpenHEARTSMAP website was also examined through websites (clicking the OpenHeartsmap.ca website link through a different website), direct entry, search engines or Twitter.

Exploratory Outcomes - Implementation, Reach and Adoption

We assessed implementation, exploring the most effective manner to use Twitter as a KT tool (hashtag versus tagging), by measuring Twitter interaction metrics of ‘engagements’ (total number of times when a user interacts with a Tweet including: clicks anywhere on the Tweet, including retweets, replies, follows, likes, links, cards, hashtags, embedded media, username, profile photo or Tweet expansion) for each Tweet.

Reach was measured for each Tweet using the metric ‘impressions’ (total views from appearing in news feeds or search results).

Adoption of OpenHEARTSMAP was quantified by the number of online registrations and forms completed of the OpenHEARTSMAP tool.

Data analysis

Primary Outcome

For measuring awareness, the mean baseline number of ‘page visits’, ‘visit duration,’ and ‘page actions per visit’ to OpenHEARTSMAP were collected for one week prior to the promotion and at the completion of the one-week promotion. Percent changes were calculated. The change in method of access to OpenHEARTSMAP was also compared between the control week and Twitter week. Changes to the primary outcome metrics were analyzed using a paired sample t-test. Normally distributed continuous explanatory variables were described using parametric tests. Results were considered statistically significant with p values < 0.05. All statistical analyses were performed using RStudio (RStudio, Boston, MA, USA).

Exploratory Outcomes

For implementation, each Tweet was ranked according to the sum number of ‘engagements’ within each category after the one-week promotion to identify the most popular KT Twitter strategy (hashtags or generic Tweet with tagged users) and topic category that enabled maximal user interaction.

For reach, the ‘impressions’ were summed per arm to grossly identify which Twitter KT strategy (hashtags or tagging) led to the largest reach.

For adoption, the mean baseline number of online registrations and forms completed for one week prior to the promotion and at the completion of the one-week promotion. Percent changes were calculated. The change in method of access to OpenHEARTSMAP was also compared between the control and Twitter weeks and taken into account.

Ethical considerations

Approval was obtained by the University of British Columbia Children and Women’s Research Ethics Board (H17-00748).

## Results

Primary outcome

**Figure 1:**
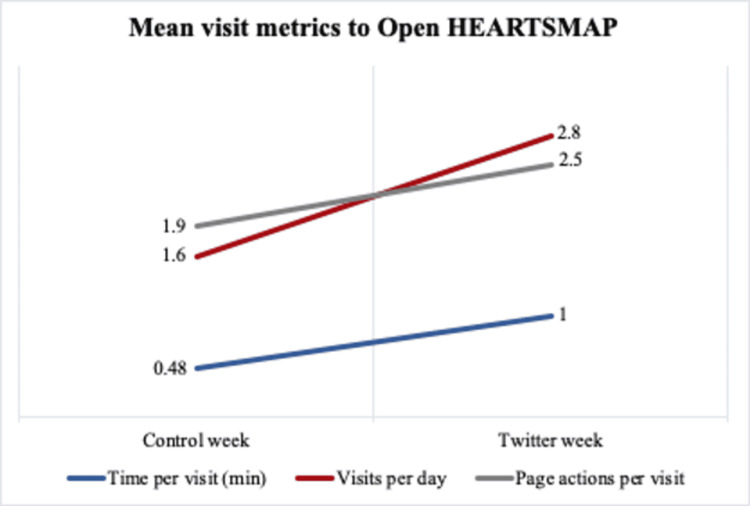
Comparison of mean time per visit (mean increase: 0.52 min; 95% CI: -0.45-1.5), mean visits per day (mean increase: 1.2; 95% CI: -0.9-3.1) and mean page actions per visit (mean increase: 0.6; 95% CI: -1.4-2.6) to the openHEARTSMAP website between the control week and Twitter week.

During the control week, an average of 1.6 visits per day was recorded on the OpenHEARTSMAP website, for a total of 13 visits (Figure 1). Visits increased by 175% during the Twitter week to an average of 2.8 visits per day, for a total of 22 visits (mean increase: 1.2; 95% CI: -0.9-3.1). There was a 212% increase in the mean time spent on the website per visit (mean time per visit for control week: 0.48 min; mean time per visit for the Twitter week: 1.00 min; mean increase: 0.52 min; 95% CI: -0.45-1.5), and a 130% increase in mean page actions per visit (mean page actions for control week: 1.9; mean page actions for the Twitter week: 2.5; mean increase: 0.6; 95% CI: -1.4-2.6).

**Figure 2:**
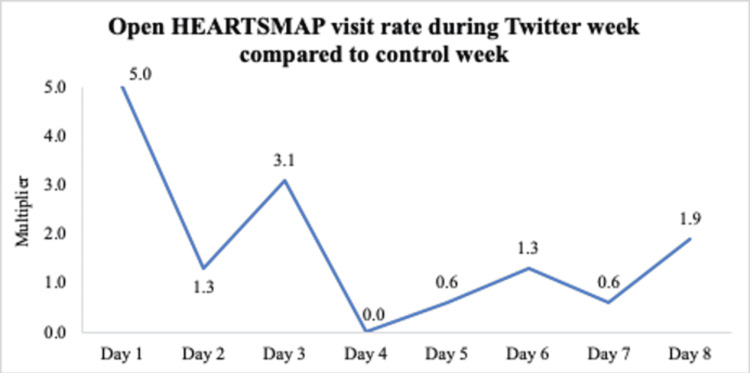
Difference in visits to the Open HEARTSMAP website per day during the Twitter posting week compared to the mean visits per day (1.6) during the control week.

Figure 2 shows the difference in visits per day to OpenHEARTSMAP compared between the Twitter week and to the average number of visits per day during the control week. The greatest increase in visits occurred on the first day of Twitter posting, with fluctuating changes on subsequent days.

**Figure 3:**
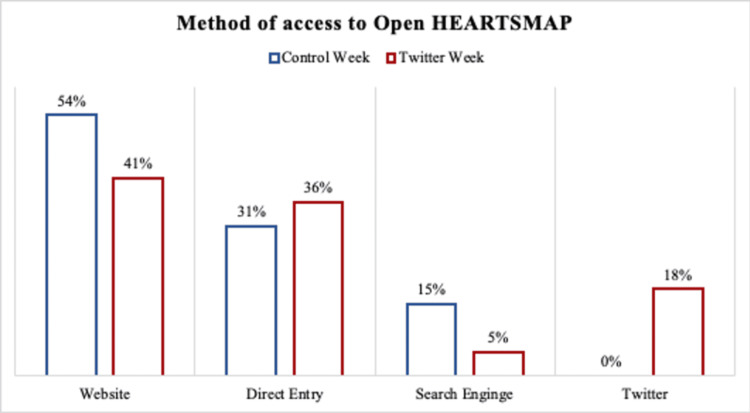
Method by which the openHEARTSMAP website was accessed in the control week compared to the Twitter posting week.

In terms of method of access, we observed a decrease in 10% or more in access through websites or search engines in favour of Twitter during the Twitter week compared to the control week (Figure 3).

Exploratory outcomes

**Figure 4:**
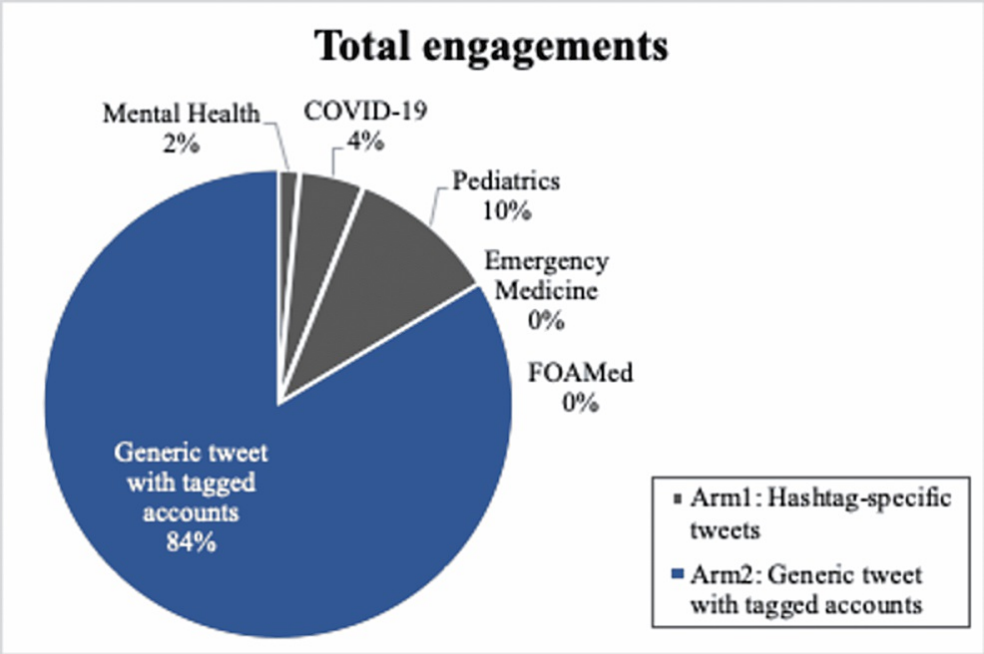
Rate of total engagements per study arm and per hashtag topic.

**Figure 5:**
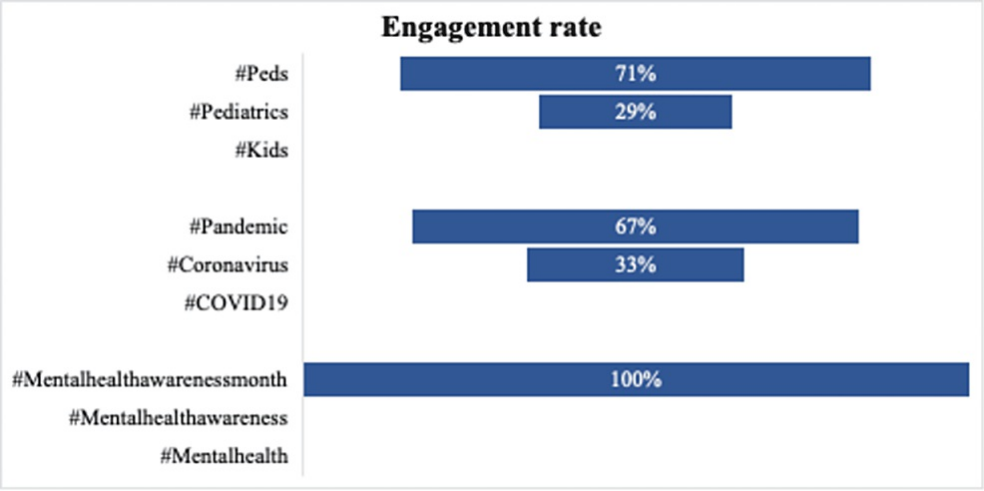
Engagement rate per hashtag topic category in study arm 1 that showed overall engagement.

In regards to impressions, Study arm 2 received greater engagements. Within arm 1, the topic category of pediatrics received the most engagements, with the hashtag #Peds as the most popular (Figures 4, 5). The topic categories of emergency medicine and FOAMed received no engagements. The sources of retweets per study arm are depicted in Table 1. None of the 15 tagged users in study arm 2 retweeted our generic post.

**Table 1:** Comparison of retweet sources in study arms. Zero of 8 retweets in study arm 2 arose from any of the 15 tagged Twitter accounts.

Retweets	
Arm1: Hashtag-specific Tweets Total retweets	1
User is our follower	100%
User is not our follower	0%
Arm2: Generic Tweet with tagged accounts Total retweets	8
User is our follower	38%
User is not our follower	62%

**Figure 6:**
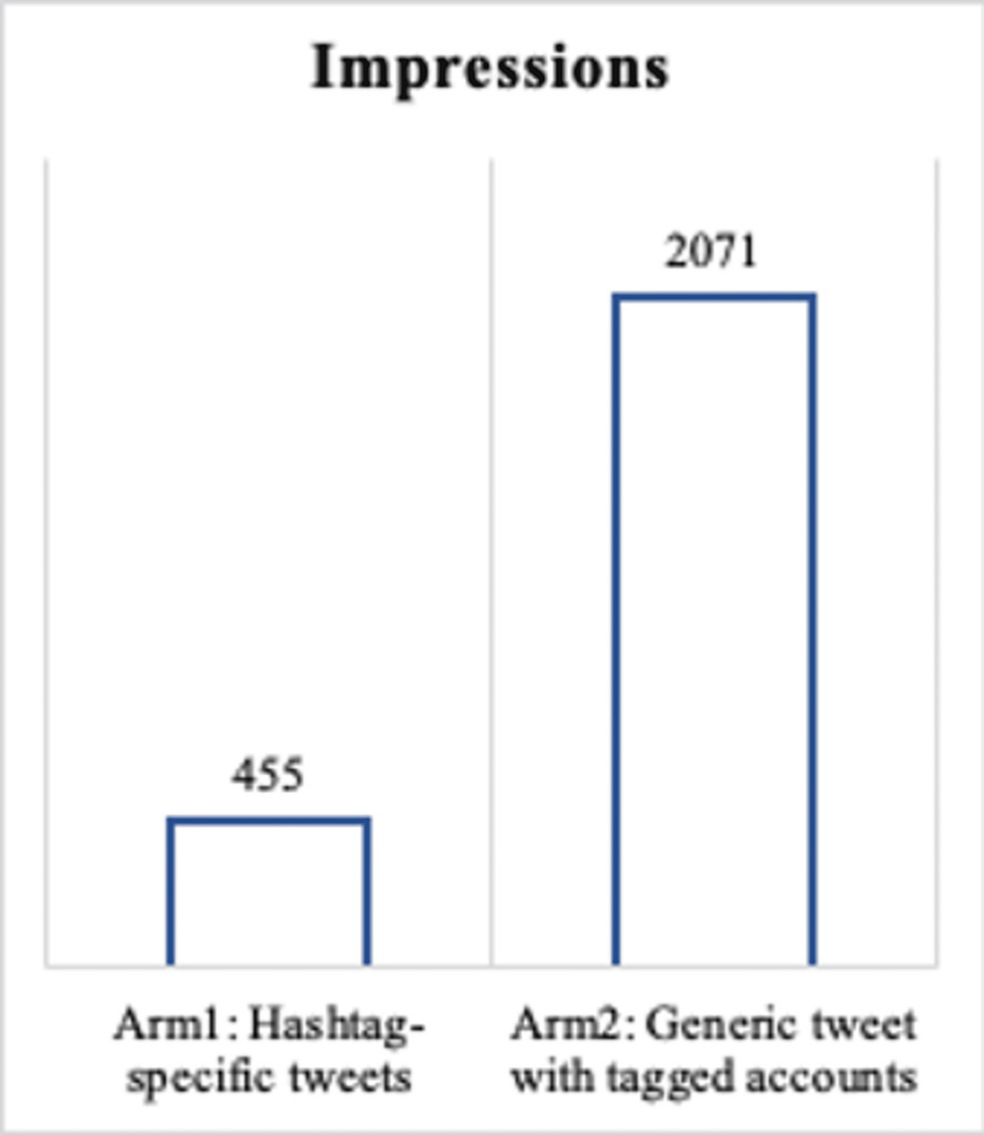
Sum of impressions per study arm.

In regards to reach, a total of 455 impressions were obtained in arm 1 compared to 2071 in arm 2 as depicted in Figure 6.

In regards to adoption, one new registration was obtained during the control week whereas no new registrations on the OpenHEARTSMAP website were obtained during the Twitter week. No new forms were started or completed during the control week nor the Twitter posting week.

## Discussion

No previous research has examined the role of social media specifically for knowledge translation in mental health as it relates to PEM. Twitter has been shown to improve knowledge of research evidence in other domains as outlined below. This is the first study, to our knowledge, to suggest that Twitter as a KT tool can be effective in increasing awareness of research in the field of PEM, the first step in KT, but not necessarily adoption, the last step in KT.

Twitter and Facebook were evaluated by Tunnecliff et al. for their efficacy as an educational platform [6]. In this study, clinicians learned about tendinopathy management via either Twitter or Facebook. Between the two groups, almost three-quarters of respondents indicated the social media intervention changed their practice and promoted increased use of research evidence in clinical practice via social media [6]. However, they noted that Twitter was more effective when building a network and disseminating information. Twitter has also been shown to increase views of medical articles and journal websites [6,7]. These studies demonstrate the general effectiveness of Twitter to disseminate knowledge to practitioners. Our study extends these findings to pediatric emergency care and pediatric mental health presentations in the ED.

Study implications

During the Twitter dissemination week, substantial increases were shown in all metrics used to measure the change in awareness - ‘page visits’, ‘average visit duration’, and ‘page actions’ on the OpenHEARTSMAP webpage. Our results confirm, with those of earlier studies, that Twitter appears to be an effective method for clinical KT to increase awareness of PEM-related research. We also found that during the Twitter dissemination week, the number of people accessing the OpenHEARTSMAP website via the Twitter link generally increased. This lends further insight into the potential of Twitter as an effective avenue to capture end-users for KT.

We measured implementation using the Twitter interaction metrics of engagements for each Tweet. Engagement is known to be a more credible and reliable measure than impressions to evaluate social media strategies. Twitter is a platform that works best with targeted content and this is supported by our findings [26]. We observed greater engagements in arm 2 where users were tagged in a message that was addressed to them directly and would have been notified by Twitter. The arm 2 Tweet was also the first Tweet posted at 09:00 PST, which may have contributed to its high engagement.

From the 15 tagged opinion leaders we identified, none (even those known personally to the research team) headed the call to action to retweeted our post. Of the eight retweets, more than half were from non-followers of the @HEARTSMAP_ account, yet still came across our Tweet from those who do follow us and retweeted. These findings support the notion of Twitter as an effective dissemination tool through a snowball effect, where one may not need to be personally connected to users in order to disseminate a clinical idea. This presents the notion that building a robust and engaged Twitter following might be helpful in clinical KT, more so than actively seeking out users to retweet a post.

Within study arm 1, the topic category of pediatrics received the most engagement (#Peds as the most popular) followed by topic categories COVID-19 and mental health. The topic categories of emergency medicine and FOAMed received no engagements. These findings suggest that using relevant hashtags coinciding with local or current events may be an effective engagement strategy (e.g., COVID-19 in May of 2020 or May being mental health awareness month). Furthermore, using the highest trending hashtag within a topic was less effective than employing hashtags that are trending within a topic, yet not the most used. This could potentially be explained by the fact that the general popularity of highly used hashtags results in rapid dilution of any single Tweet.

Reach was measured for each Tweet using impressions. Arm 2 of our study had more than four and a half times as many impressions. Despite these results, it cannot be concluded that tagging users in a Tweet is a more successful strategy for KT. For one, this Tweet for arm 2 happened to be the first Tweet that was posted at 09:00 PST, which, based on parallel data from the OpenHEARTSMAP website visit times, generally marks a higher traffic time which also coincides with the highest healthcare-related traffic times [27]. This could be a factor in the larger number of impressions for the Tweet in arm 2. Alternatively, this Tweet also received more engagements (including the 15 comments we made to tag users), which could have made it more likely to appear higher in a followers' news feeds due to algorithms that preferentially depict more relevant content rather than content in chronological order. While more impressions may seem better, the metric might be misleading [27]. Impressions cannot guarantee that users have truly seen or interacted with the Tweet. For this reason, engagements are a more accurate reflection of interaction [26].

Adoption, or use, of OpenHEARTSMAP was quantified by the number of online registrations and forms completed of the OpenHEARTSMAP tool. There was no change in the number of completed forms between the control week and the Twitter dissemination week and one new registration on the OpenHEARTSMAP website during the control week. While adoptability, or behavioural change, is the ultimate measure of successful KT, this study focused on awareness as the primary measure of KT, therefore these results do not take away from our findings of Twitter as an effective KT tool to increase awareness of PEM research. Our findings are comparable to Hawkins et al. who explored whether social media could increase journal subscriptions and readership [28]. They discovered that social media was a fruitful tool for increasing awareness, though had little to no impact on adoptability of readership. Our findings, though similar, are novel in the field of PEM mental health. Like readership in their study, the use of our OpenHEARTSMAP tool is a subjective decision. This reflects the attitude and behaviour-related domains as the second and third steps respectively in the pipeline of effective KT beyond the scope of this study.

Limitations

While our primary objective was to drive traffic to our website using Twitter, the small numbers in this study preclude us from drawing definitive associations between Twitter and website metrics which have previously been suggested to correlate poorly [29]. Furthermore, due to a lack of available methods, we were unable to identify which Tweets or which study arm, in particular, led to the increase in website visits to further elucidate the social media strategy on Twitter that was most effective. Finally, it is possible that our findings were confounded by parallel training of OpenHEARTSMAP outside of social media in March to early May 2020, in keeping with local clinical efforts during the COVID-19 pandemic. However, this confounder was present across both the control and Twitter weeks. Future Twitter-based studies in PEM mental health could consider the above limitations, incorporate advanced metrics such as the rate of retweets, control for the effect of time of day of posts as well as elucidate strategies for changing attitudes and increasing adoption of research, being the second and third steps of KT.

## Conclusions

Twitter can be an effective KT tool to increase awareness of research, the first step of KT, in the domain of PEM mental health care. During our Twitter promotion, there were increases in visits, mean time spent and mean page action per visit to the OpenHEARTSMAP webpage. Strategies for successful dissemination via Twitter derived from this study include building a robust following; posting during peak healthcare-related Twitter traffic times; employing hashtags coinciding with current events rather than employing the most trending hashtags; and targeting posts by tagging users, who need not necessarily be generally known opinion leaders.

## Appendices

**Table 2:** OpenHEARTSMAP Question Guide

HOME	• Is there difficulty or fighting at home between family members? • How do you get along with [guardian/parents/family]? • How do you feel about your home environment?									
Assessment Notes	No concerns	Mild Concerns		Moderate Concerns					Major Concerns	
	0	1		2					3	
		Supportive of youth's difficulties but some conflicts.		Unsupportive (parents at risk for burn out). Frequent conflicts.					Dysfunctional (parental burn out). Homelessness. Major conflicts.	
	o	o		o					o	
	Resources: Social Supports or family counseling services neither requested nor initiated. Social Supports or family counseling involved (resource requested and services initiated)									
EDUCATION & ACTIVITIES	• How is school going for you? • Are there any difficulties going to school or staying in class? • What do you do for fun? Has that changed recently?									
Assessment Notes	No concerns	Mild Concerns				Moderate Concerns		Major Concerns		
	0	1				2		3		
		Struggle to maintain. Difficulty attending. Attends more than misses				Performance decline. Missing classes/activities. Misses more than attends.		Failing / major issues. Not attending. Completely truant (excluding holidays)		
	o	^.^o				o		o		
	Resources: Educational/Activity issues not yet addressed. Functional Plan in place (counselor or school authorities involved)									
ALCOHOL & DRUGS	• How much is alcohol use a part of your life? • Do you use any substances like marijuana? How about any others? • Do you ever use drugs or alcohol to feel better or to make a problem go away?									
Assessment Notes	No Concerns		Mild Concerns				Moderate Concerns	Major Concerns		
	0		1				2	3		
			Infrequent. Mild recreational use.				Regular recreational use. Mild substance misuse	Binging recreational use. Substance abuse.		
	o		o				o	o		
	Resources: No substance use services or no referral to substance use services in place. Substance use services or program in place (referred or offered)									
RELATIONSHIPS & BULLYING	• How are things going for you with friends and relationships? • Do you have a close person/group of people that you can rely on? • Do you feel teased, bullied, or excluded by others? • Do you have any struggles with your sexual identity or sexual preference?									
Assessment Notes	No Concerns	Mild Concerns				Moderate Concerns		Major Concerns		
	0	1				2		3		
		Minor conflicts / bullying. Struggle to maintain.				Conflicts / bullying. Negative changes.		Major conflicts / bullying. Lack of relationships. Major dysfunctional relationship.		
	o	o				o		o		
	Resources: No support or resources initiated. Educational or social plan in place (school authority or social worker aware and addressing)									
THOUGHTS & ANXIETY	• Do you consider yourself someone who worries or thinks a lot about the past or future? • Do you ever experience panic / extreme fear that comes out of the blue? • Do you hear voices or see things that aren’t there or aren’t real? • Do you believe you have special powers or receive special messages? • Do you ever have times where you feel your brain is playing tricks on you? • Do you generally feel safe?									
Assessment Notes	No Concerns	Mild Concerns			Moderate Concerns					Major Concerns
	0	1			2					3
		Anxiety / odd thoughts (minimal impact).			Moderate anxiety or thought problems (strong, but able to power through).					High anxiety (impairing / insurmountable). Thought disorder / psychosis.
	o	o			o					o
	Resources: No psychiatric assessment or services initiated yet (not yet referred or on wait list for initial assessment and no appointment in sight). Care plan in place (Local community mental health services, urgent response team, psychiatrist, or private counselor/psychologist involved or will be involved shortly, and available in the long term irrespective of youth's adherence)									

SAFETY	• Do you sometimes feel hopeless, or that life is not worth living? • In the past few weeks, have you seriously considered ending your life or ending someone else’s life? • Have you ever tried to end your life or someone else’s life? (If Yes:) How did you try to end your life or someone else’s life? • In the past few weeks, have you thought of harming yourself or others? • In the past few weeks, have you felt that you or your family would be better off if you were dead?										
Assessment Notes	No Concerns	Mild Concerns					Moderate Concerns			Major Concerns	
	0	1					2			3	
		Fleeting or improving thoughts. Non-suicidal self injury. Verbal threats to others but no action.					Passive suicidal ideation. Non-lethal gestures to self (suicide practicing) or others.			Formed plan. Lethal gestures to self or others. Attempt.	
	o	o					o			o	
	Resources: No plan for current safety concern. Safety planning in place AND consistent with current suicidality/homicidality										
SEXUAL HEALTH	• Are you involved in any sexual activities / not limited to penetration? • Do you use any mode of contraception? • What form of protection against sexually transmitted disease do you use if any? • Do you get any counseling about sexual health from a doctor or nurse?										
Assessment Notes	No Concerns	Mild Concerns		Moderate Concerns					Major Concerns		
	0	1		2					3		
		Sexually active and safe practice (contraception and STD protected).		Stable partner but inconsistent use of protection and contraception.					Multiple partners or no use of protection or contraception. Involved in sex trade.		
	o	o		o					o		
	Resources: Sexual health issues not yet approached with health care professional. Has a primary care provider and issues of sexual health/family planning addressed										
MOOD	• How would you rate your mood, with '0' being as low as possible, and '10' being perfectly happy? • Do you feel down or depressed recently? • Do you feel really happy or energetic lately?										
Assessment Notes	No Concerns		Mild Concerns			Moderate Concerns					Major Concerns
	0		1			2					3
			Mood instability (minor). A few concerning behaviours.			Depression / irritability. Concerning behaviours.					Severe depression / manic. Major behavioral concern.
	o		o			o					o
	Resources: No psychiatric assessment or services initiated yet (not yet referred or on wait list for initial assessment and no appointment in sight). Care plan in place (Local community mental health services, urgent response team, psychiatrist, or private counselor/psychologist involved or will be involved shortly, and available in the long term irrespective of youth's adherence)										
ABUSE	• To child: Has anyone ever hurt you by touching you in a way you didn't like? • To adolescent: Have you ever experienced abuse, either physical, emotional, or sexual? • To caregiver: Do you have any concerns of abuse or mistreatment?										
Assessment Notes	No Concerns		Moderate Concerns								Major Concerns
	0		2								3
			Concern has been raised and reported to local child protection services. Historical concerns. At risk for grooming / victimization.								Current concern of abuse or neglect / not reported.
	o		o								o
	Notification has occurred: o Yes o No										
PROFESSIONALS & RESOURCES	• Do you feel that there are people or places you can go to for help? • Who are the people who are working with you on these issues? • Does the current plan to help make sense to you?										
Assessment Notes	No Concerns				Moderate Concerns			Major Concerns			
	0				2			3			
	Service plan in place or available. No new outpatient or long term referrals need to be made.				Referred for service, but access delayed (wait listed).			Longitudinal services unavailable, but necessary. Not yet referred or refusing services/treatment.			
	o				o			o			
